# Discovery, screening and evaluation of a plasma biomarker panel for subjects with psychological suboptimal health state using ^1^H-NMR-based metabolomics profiles

**DOI:** 10.1038/srep33820

**Published:** 2016-09-21

**Authors:** Jun-sheng Tian, Xiao-tao Xia, Yan-fei Wu, Lei Zhao, Huan Xiang, Guan-hua Du, Xiang Zhang, Xue-mei Qin

**Affiliations:** 1 Modern Research Center for Traditional Chinese Medicine of Shanxi University, Taiyuan 030006, P. R. China; 2 College of Chemistry and Chemical Engineering of Shanxi University, Taiyuan 030006, P. R. China; 3 Department of traditional Chinese medicine, First Hospital of Shanxi Medical University, Taiyuan 030001, P. R. China; 4 Physical Education Departments of Shanxi University, Taiyuan 030006, P. R. China; 5 Institute of Materia Medica, Chinese Academy of Medical Sciences & Peking Union Medical College, Beijing 100050, P. R. China; 6 Department of Chemistry of University of Louisville, Louisville, KY40292 United States

## Abstract

Individuals in the state of psychological suboptimal health keep increasing, only scales and questionnaires were used to diagnose in clinic under current conditions, and symptoms of high reliability and accuracy are destitute. Therefore, the noninvasive and precise laboratory diagnostic methods are needed. This study aimed to develop an objective method through screen potential biomarkers or a biomarker panel to facilitate the diagnosis in clinic using plasma metabolomics. Profiles were based on H-nuclear magnetic resonance (^1^H-NMR) metabolomics techniques combing with multivariate statistical analysis. Furthermore, methods of correlation analysis with Metaboanalyst 3.0 for selecting a biomarker panel, traditional Chinese medicine (TCM) drug intervention for validating the close relations between the biomarker panel and the state and the receiver operating characteristic curves (ROC curves) analysis for evaluation of clinical diagnosis ability were carried out. 9 endogenous metabolites containing trimethylamine oxide (TMAO), glutamine, N-acetyl-glycoproteins, citrate, tyrosine, phenylalanine, isoleucine, valine and glucose were identified and considered as potential biomarkers. Then a biomarker panel consisting of phenylalanine, glutamine, tyrosine, citrate, N-acetyl-glycoproteins and TMAO was selected, which exhibited the highest area under the curve (AUC = 0.971). This study provided critical insight into the pathological mechanism of psychological suboptimal health and would supply a novel and valuable diagnostic method.

A number of individuals are struggling with the category of low-quality-status medically unexplained symptoms (MUSs)^1^ and the morbidity rate is 1.6–70%, 2.4–87% and 4.6–18% in young, middle aged and elderly populations dividually from 1966 according to the investigation of MUSs^2^. Meanwhile, MUSs has been defined as “suboptimal health” (*Yajiankang* in China) in traditional Chinese medicine explained as a borderline state between health and disease. To our disappointment, suboptimal health is more difficult to be diagnosed with a biological disease because of only vague changes in function but no clear signs of organic disease^3^, which present as low energy level, loss of vitality, altered sleeping patterns and so on^4^. It could be parallel with symptoms of chronic fatigue syndrome (CFS)^5^,^6^ or “THE THIRD STATE” or “GRAY STATE” raised by the former Soviet scholar prospectively. Also state of suboptimal health included several different subtypes, and as a subtype, psychological suboptimal health has attracted more attentions.

Psychological suboptimal health is a prevalent state with a pathophysiological mechanism that is extremely complicated and poorly understood. Although it exhibits objective symptoms without a specific disease and it cannot reach the standards of psychiatric diagnosis such as depression and anxiety neurosis estimated by scores on diagnostic scales, the 10th edition of international Classification of diseases (ICD-10), Classification and Diagnostic Criteria of Mental Disorders in China-Third-Edition (CCMD-3), the 4th edition Diagnostic and Statistical Manual of Mental Disorder (DSM-IV)^7^, for instance, but we must not ignore potential hazards. As the intermediate state between mental health and psychological disease, the emblematical symptoms indicating someone immersed in the state contain out of humor, panic, negative emotion, easy to get angry, losing interest, insomnia, impaired concentration and so on. What’s more, psychological suboptimal health, can result in crippling quality of life and raising costs in medical due to frequent, unnecessary visits to healthcare facilities for checkups and diagnoses.

In virtue of potential damage and ambiguity in pathomechanism, psychological suboptimal health has garnered increasing attention and has been described in experimental reports and defined as “subthreshold depression”^8^,^9^ or “subthreshold obsessive-compulsive disorder”^10^, the concepts of which are very similar to psychological suboptimal health. In addition, Blackwood has divided chronic fatigue syndrome (CFS) into two states of psychological and physical in the survey^11^ and the psychological state of CFS is parallel to psychological suboptimal health. These researchers laid particular emphasis on epidemiologic characteristics, and unfortunately, studies on psychological suboptimal health and the pathogenetic mechanisms involved are rare relatively. What’s more, a diagnostic criterion that effective and widely accepted has not been established at home and abroad. In China, scholars and doctors prefer to use a variety of scales and questionnaires to diagnose the intermediate state, including the Symptom Checklist 90 (SCL-90), Cornell Medical Index (CMI), mental functions decline index health assessment (MDI), or other self-made evaluations, combining with subjective judgment. To a certain extent, these approaches are authentic for diagnosis but at the same time, rate of missed diagnosis and misdiagnosis is not satisfying, owing to inconformity indigestibility of scales, concealment of patients, doctors relying too much on experiences and subjective judgment. So diagnostic methods that objective, high reliability and easy to operate need to be developed urgently. Through the approach of evaluating the significant differences at the molecular level, novel biomarkers or a biomarker panel then further could be discovered in the plasma samples from patients with psychological suboptimal health and healthy controls. And they would be used in clinical diagnosis after further validation and evaluation. Moreover, metabolomics technologies are the principal approaches for diseases biomarkers discovering.

Systems biology^12^ including genomics, proteomics, and metabolomics can be utilized in research of diseases^13^,^14^,^15^. As an important component of systems biology, metabolomics technologies have become a powerful tool and platform for detecting endogenous small compounds^16^,^17^ as candidate biomarkers closely related to pathological and physiological processes of diseases and carrying rich information concerning metabolism as key pathways^18^. It may help to unravel the mechanisms of disease occurrence and progression on the metabolic level^19^. Also major metabolomics technologies were based on H-nuclear magnetic resonance (^1^H-NMR)^20^,^21^,^22^, liquid chromatography−mass spectrometry (LC−MS)^23^, and gas chromatography−mass spectrometry (GC−MS)^24^,^25^. Furthermore, it was noteworthy that ^1^H-NMR is the earliest method used in metabolomics analysis with the advantages of possessing a rapid, non-destructive, high-throughput system^26^, and still is widely used to detect biomarkers of diseases for clinical diagnosis^27^.

In common sense, medicine should be applied to improve clinical symptoms, but few chemical drugs was suitable. As a well-known traditional Chinese prescription, Baihe Dihuang Tang (BDT) is described initially in “Synopsis of Golden Chamber” (Jinkui *Yaolue*) consisting of two herbal medications: lily bulb (Bulbus Lilii) and rehmannia root (Radix, Rehmanniae). It is used to treat mental instability, absentmindedness, insomnia, and dysphoria in clinical. These major symptoms are closely associated with early depression disorder^28^ and also perform in psychological suboptimal health state. Furthermore, BDT has been widely used and significantly improved the symptoms of psychological suboptimal health due to a deficiency of *yin (Yin Xu*), according to the theory of TCM and also BDT was applied as the intervention measure in our experimentation.

As far as we know, just several published papers were involved in the research of suboptimal health state with meatabolomics and achieved some results^29^,^30^,^31^ but the study of the psychological suboptimal health state taking advantage of metabolomics technology is almost a blank. In the present study, plasma metabolomics based on ^1^H-NMR coupled with multivariate statistical analysis are used for investigating metabolites with significant differences at a molecular level and screening potential biomarkers. What the goal is to develop a biomarker panel from the biomarkers through correlation analysis, drug intervention of BDT and evaluation of diagnostic ability that can be used for clinical diagnosis ultimately. A biomarker panel would provide support for objective diagnostic laboratory tests for psychological suboptimal health.

## Results

### Clinical information of participators

According to the scale and clinical diagnosis, 22 patients being in state of psychological suboptimal health and 23 volunteers acting as the healthy control group were screened. From the SCL-90 scores of 143.9 ± 22.6 and 90 as the mean ± SD form and the filter factors mentioned above, a significant difference between two groups was confirmed in clinical. The basic clinical data for the participators are shown in Table 1.

### ^1^H-NMR spectra of plasma

**Figure 1:**
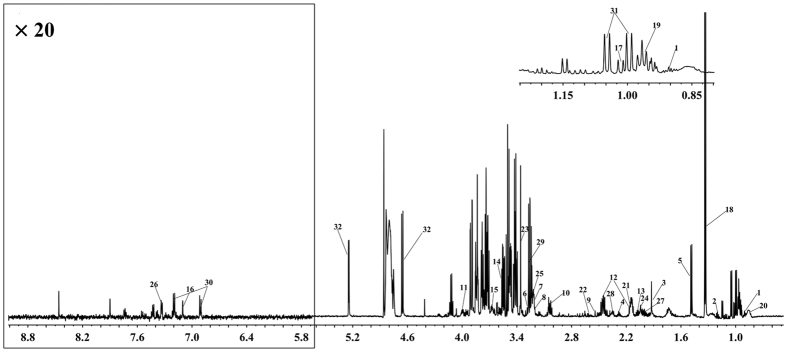
Typical ^1^H-NMR spectrum of plasma of psychological suboptimal human subject. The key identified metabolites: 1, 2-OH-butyrate; 2, 3-OH-butyrate; 3, Acetate; 4, Acetoacetate; 5, Alanine; 6, Betaine; 7, Carnitine; 8, Choline; 9, Citrate; 10, Creatine; 11, Cysteine; 12, Glutamine; 13, Glutamate; 14, Glycine; 15, Glycerol; 16, Histidine; 17, Isoleucine; 18, Lactate; 19, Leucine; 20, Lipids; 21, Methionine; 22, Methylamine; 23, Methanol; 24, N-acetyl-glycoproteins; 25, Phosphatidylcholine; 26, Phenylalanine; 27, Proline; 28, Pyruvate; 29, Trimethylamine oxide; 30, Tyrosine; 31, Valine; 32, Glucose.

To identify the small endogenous molecules in plasma and survey the level varieties in different states, all samples were processed, and typical Carr-Purcell-Meiboom-Gill(CPMG) ^1^H-NMR spectra of plasma from groups of psychological suboptimal health was depicted (Fig. 1). 32 metabolites were identified according to the Human Metabolome Database (HMDB: http://www.hmdb.ca/), the Chenomx NMR suite (Chenomx Inc, Edmonton, AB, Canada) and previously published references^32^,^33^,^34^. For a better visualization, the vertical scales for the 2D spectra, including 1H-1H correlation spectroscopy (1H–1H COSY) and 1H–13C heteronuclear multiple quantum correlation (1H–13C HMQC) spectra (Supplementary Figures S1 and S2) were adjusted based on metabolite peaks. Plasma spectra from healthy controls and the BDT group are shown in Supplementary Figures S3 and S4. The metabolites identified in the spectra were listed in Table 2. Several amino acids, glucose, organic acids, lipids, choline were demonstrated in the spectra.

### Validation and assessment of the differences between groups

**Figure 2:**
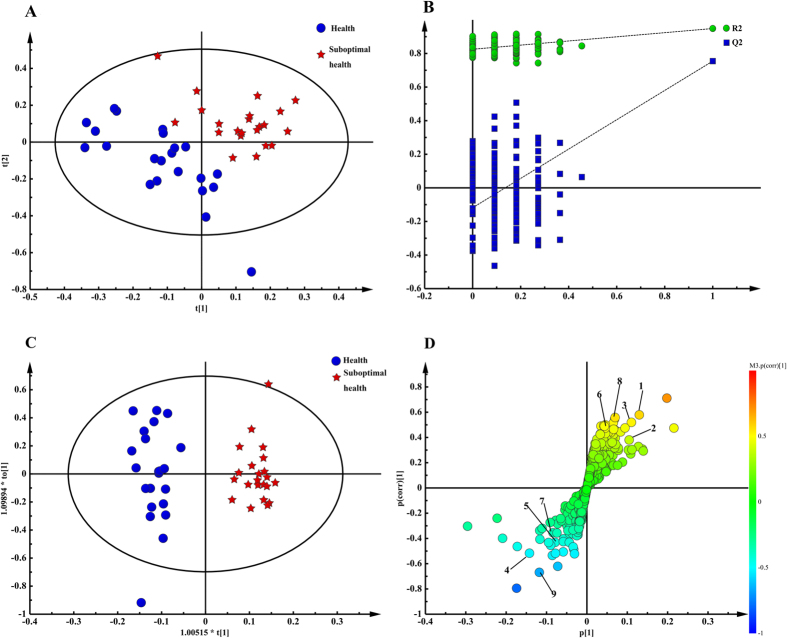
Pattern recognition with Simca-P13.0. The PLS-DA score plot derived from ^1^H-NMR plasma spectra of psychological suboptimal group compared with healthy controls (**A**). The PLS-DA validation plots (permutation number: 200) pair-wise comparison of plasma from psychological suboptimal group and healthy controls (**B**). The OPLS-DA score plot derived from 1H NMR plasma spectra of psychological suboptimal group compared with healthy controls (**C**) Corresponding S-plot between psychological suboptimal group and healthy controls and the metabolites changed significantly:1, N-acetyl-glycoproteins; 2, Trimethylamine oxide; 3, Glutamine; 4, Glucose; 5, Valine; 6, Phenylalanine; 7, Isoleucine; 8, Citrate; 9, Tyrosine (**D**).

With the purpose of demonstrating significant differences not only in the clinical scale scores, we analyzed the NMR spectra information using multivariable statistics. Metabolome difference by comparing the numerical integration was observed and partial least squares discrimination analysis (PLS-DA)-based profiling was employed to explore the intrinsic differences between the groups of psychological suboptimal health and mental health. The samples from different groups were separated and classified into two distinct clusters presented in the PLS-DA score plot (Fig. 2A); each point represents an individual sample (to show the group clusters). The model parameters (R^2^X = 0.541, R^2^Y = 0.949, Q^2^ = 0.755) and the validated model (permutation number: 200) indicated no over fitting (Fig. 2B), supporting the result. All of the results indicated the existence of differences between the two groups and the reliability of diagnosis according to the method with scales mentioned previously.

### Discovery and screening of potential biomarkers

**Figure 3:**
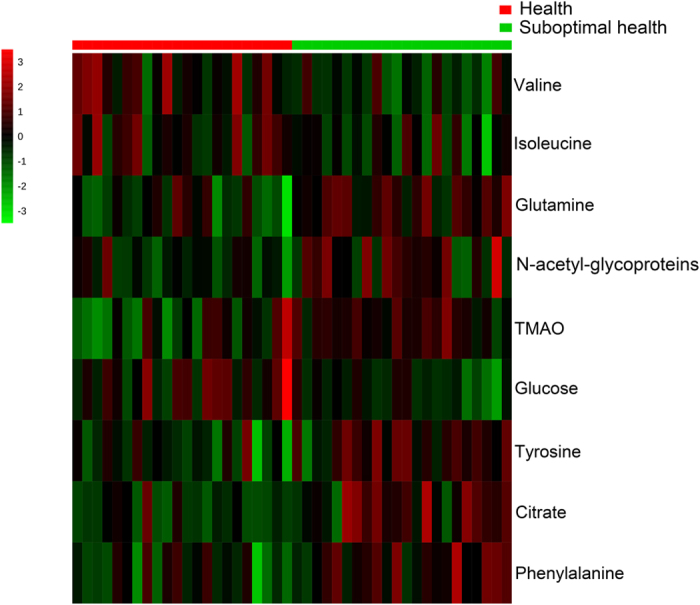
The heatmap plot between group of psychological suboptimal health and healthy controls. Red color indicates a higher level and green color indicates a lower level.

To identify changed metabolites and considering the high information content and complexity of the spectra, orthogonal partial least squares discriminant analysis (OPLS-DA) was used to amplify the subtle differences due to the abnormal state of psychological suboptimal health. The supervised model of OPLS-DA could develop a better separation into two clusters and contribute to the discovery of biomarkers. The group of psychological suboptimal health exhibited a perfect separation from the healthy controls in the OPLS-DA scores plot (Fig. 2C), as well as in permutation tests and CV-ANOVA (*p* < 0.05). All parameters indicating the model quality were listed in Supplementary Table S1. The metabolites responsible for a significant contribution to the separation of two groups were indicated in the corresponding S-plot (Fig. 2D) and marked with number containing glutamine, N-acetyl-glycoproteins, TMAO, citrate, phenylalanine, valine, isoleucine, tyrosine and glucose. The specific change trends that higher levels of glutamine, N-acetyl-glycoproteins, TMAO, citrate, tyrosine and phenylalanine and lower levels of valine, isoleucine, and glucose were revealed in Table 3. Furthermore, a heatmap plot with different color that green stands for low level and the red is opposite was constructed, from which we could observe the trends more visually (Fig. 3).

### Screening biomarker panel

#### Correlation analysis of potential biomarkers

**Figure 4:**
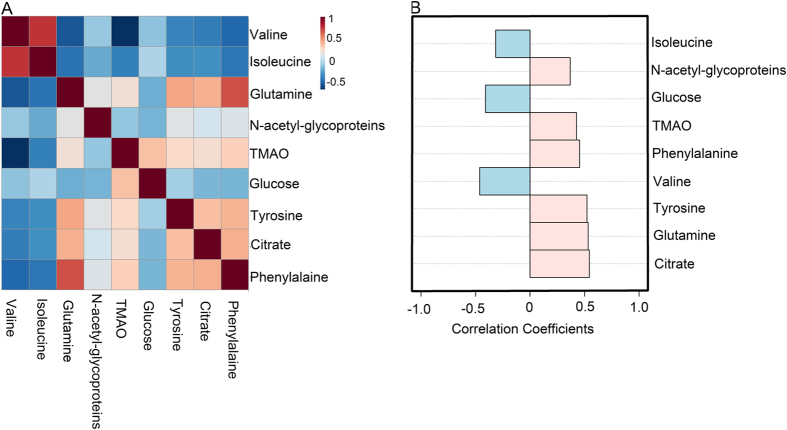
Systems analysis of potential biomarkers of psychological suboptimal and healthy controls with MetaboAnalyst 3.0 data annotation tools. The correlation heatmap display the correlation coefficients (Spearman) among biomarkers. The color-coded scale of correlation is at left, where a red color indicates a positive correlation, while a blue color indicates a negative correlation (**A**). The correlation analysis with Pattern Hunter (Spearman) between group of psychological suboptimal health and healthy controls, a red color indicates a positive correlation with the state of psychological suboptimal health, a blue color indicates a negative correlation with the state of psychological suboptimal (**B**).

To investigate the relationship among the potential biomarkers, the levels in the plasma samples from patients and healthy controls were correlated using Spearman’s correlation (Fig. 4A) with Metaboanalyst 3.0^35^, an online data tool. A positive correlation indicated the relationship of the metabolites with certain pathways that exerted influence on the state of psychological suboptimal health and was distinguished with a red color, whereas a negative correlation suggested the metabolites may be from different pathways and was indicated with a blue color^36^. Analysis of the correlation among these potential biomarkers can be used to identify a biomarker panel. Remarkably, citrate was positively correlated with phenylalanine, glutamine, tyrosine and TMAO between patients of psychological suboptimal health and healthy controls. In additional, phenylalanine levels were positively correlated with N-acetyl-glycoproteins, glutamine, tyrosine, TMAO and citrate.

Further analysis using Pattern Hunter with Spearman coefficients was applied to identify the correlation between groups of healthy control and patients. Phenylalanine, glutamine, tyrosine, TMAO, N-acetyl-glycoproteins and citrate have been demonstrated a positive correlation, whereas a negative correlation of isoleucine, valine and glucose was observed between the two groups of different groups (Fig. 4B). Correlation analysis of plasma metabolites displaying significant difference was performed to gain insight into the pathogenic characteristics and pathways involved. With a purpose of selecting biomarkers that were positively correlated with state of psychological suboptimal health and forming a biomarker panel, 6 metabolites containing phenylalanine, glutamine, tyrosine, TMAO, N-acetyl-glycoproteins and citrate were selected and defined as a biomarker panel from the 9 potential biomarkers.

#### Drug intervention and validation

Based on the significantly decreased frequency of clinical symptoms and scores of SCL-90 after treatment for 4 weeks (*P* < 0.05), BDT exerted an obvious effect on improvement of symptoms, and 22 patients in state of psychological suboptimal health improved markedly These results are shown in Table 1.

**Figure 5:**
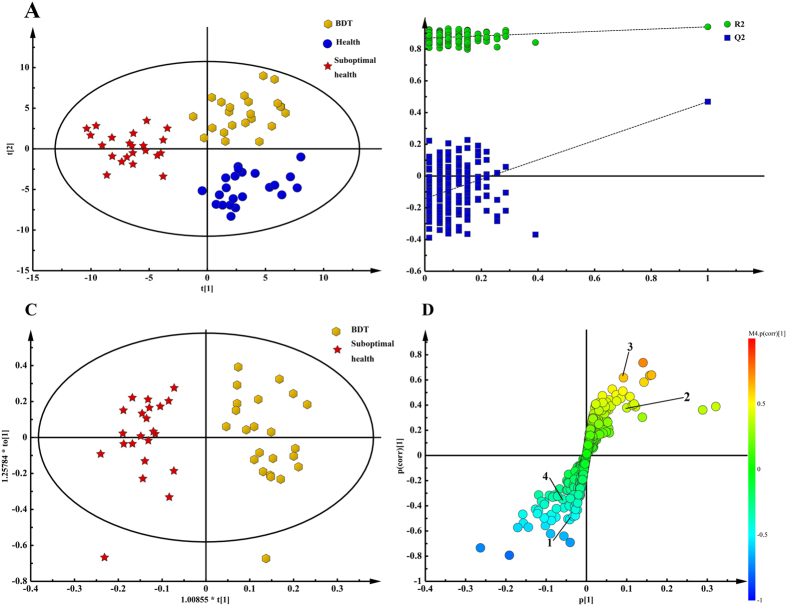
Pattern recognition with Simca-P13.0. The PLS-DA scores plot derived from all the ^1^H-NMR plasma spectra including healthy controls, psychological suboptimal group and BDT group (**A**). The PLS-DA validation plots (permutation number: 200) for all samples including healthy controls, psychological suboptimal group and BDT group (**B**). The OPLS-DA scores plot between psychological suboptimal group and BDT group (**C**). Corresponding S-plot between psychological suboptimal group and BDT group and the metabolites changed significantly: 1, Phenylalanine; 2, Trimethylamine oxide; 3, Valine; 4, Glutamine (**D**).

To obtain an overview of the metabolic responses to the actions of BDT, the PLS-DA (R^2^X = 0.15, R^2^Y = 0.941, Q^2^ = 0.531) trajectories (Fig. 5A) of all of the spectra from plasma samples containing healthy controls, pre- and post-BDT-treated groups were analyzed and separated into three clusters as outstanding differentiation. In the scores plot, the BDT treatment group was close to the healthy control group. The trend of transformation suggested the disturbance of the plasma metabolic profile of patients and stabilization after BDT administration. The validated model indicated no over fitting (Fig. 5B).

Using the strategy mentioned previously, as could be observed in the PLS-DA scores plot (R^2^X = 0.403, R^2^Y = 0.894, Q^2^ = 0.687) (Figure S5A) and the validated model that indicated no over fitting (Figure S5B), the psychological suboptimal health group and the BDT-treatment group were clearly seen as separated. The OPLS-DA model (Fig. 5C) and corresponding S-plot (Fig. 5D) indicated that the levels of the potential biomarkers tended to recover to a normal level. The levels of valine, glutamine, TMAO and phenylalanine changed significantly and reversed to normal levels after BDT treatment (*P* < 0.01, *P* < 0.05). And the metabolites changed significantly mentioned above were labeled with number (Fig. 5D). The *t*-test results of significant differences in these potential biomarkers and their changes after BDT administration are shown in Table 3. Permutation tests and CV-ANOVA (*p* < 0.05) were also performed. All parameters indicating the model quality are listed in Supplementary Table S1.

As a result, BDT treatment showed the obvious effect on the biomarker panel that levels of glutamine, TMAO, and phenylalanine that changed significantly and also citrate, tyrosine and N-acetyl-glycoproteins exhibited a trend to normal levels. As a TCM for treating mental and emotional diseases, BDT drug intervention could demonstrate the high correlation between the biomarker panel and pathomechanism of psychological suboptimal health to a limited extent.

### Diagnostic capability evaluation of biomarker panel

**Figure 6:**
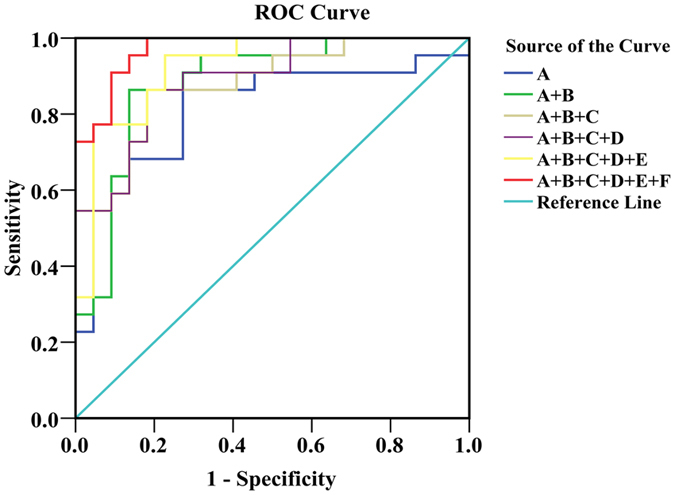
The ROC curves of different biomarker combinations for diagnosis between group of psychological suboptimal and healthy controls. A, Citrate; B, Glutamine; C, Tyrosine; D, Phenylalanine; E, TMAO; F, N-acetyl-glycoproteins.

Biomarkers with higher sensitivity and specificity are expected to be developed. ROC analysis was applied to evaluate the accuracy of diagnosis based on the identified potential biomarkers or combinations and the area under the curve (AUC) of ROC; 0.5 < AUC < 0.7, 0.7 < AUC < 0.9, AUC > 0.9 explain a low, fair, and superior accuracy of diagnosis, respectively. For most of the biomarkers, AUCs were <0.8 (Supplementary Figure S6 and Table S2), indicating a poor prediction probably in virtue of the inability of a single metabolite to predict a disease accurately or a small sample size. By selecting the metabolites demonstrating an AUC > 0.7, some combinations of potential biomarkers, including the biomarker panel mentioned above that could provide higher predictive power than single one, were examined. Finally, the AUC of the biomarker panel reached 0.971. The ROC curves and AUCs of the combinations are shown in Fig. 6 and Table 4. The AUC of the biomarker panel containing 6 metabolites indicated the highest predictive ability and the highest correlation with psychological suboptimal health.

In this study, methods of statistical analysis, correlation analysis, drug intervention and the ROC analysis were united, and a biomarker panel tightly correlated with psychological suboptimal health was identified and demonstrated.

Combined with all the analysis, these findings revealed that the biomarker panel is reliable and robust and possess a perfect ability to separate psychological suboptimal health. In future, it would be a better diagnostic approach in clinical.

## Discussion

As we have known, few studies focus on establishing an objective and accurate diagnostic method of psychological suboptimal. Scales and questionnaires in public or self-made are applied in clinic widely, whereas an more credible standard of diagnosis has not been formulated yet. The existing circumstances of lack of objective laboratory diagnosis for early detection and curative effect evaluation index may bring about adverse effects in disease prevention such as depression or. As an exploration, this study applied NMR metabolomics in investigating the state of psychological suboptimal health that meaning “not yet ill” for the first time with the purpose of seeking out potential biomarkers or a biomarker panel highly related with the state and setting it as a laboratory diagnostic method in clinical.

In this study, we discovered that a set of altered metabolites including amino acid (isoleucine, valine, phenylalanine, glutamine, and tyrosine), energy metabolism-related molecules (citrate and glucose) and other metabolism molecules (N-acetyl-glycoproteins and TMAO) that would be the potential biomarkers. A deeper insight of the internal relationship among the potential biomarkers and metabolic mechanisms closely related with state of psychological suboptimal should be gained and biological significance of potential biomarkers should be analyzed. We constructed systematic metabolic pathway analysis based on information obtained from the Kyoto Encyclopedia of Genes and Genomes Web site (www.genome.jp/kegg/) and would be discussed in further details below.

As a mental and emotional disorder, the out of control metabolic pathway highly interrelated with the state of psychological suboptimal health may relate with nervous system. And some perturbed significantly metabolites involved in neurotransmission including phenylalanine, tyrosine, valine and isoleucine were observed indeed. Phenylalanine is an essential amino acid absorbed from food that can be metabolized primarily in the liver into tyrosine, which is then used in dopamine (DA), norepinephrine (NE) and epinephrine synthesis in the nervous system and the adrenal medulla^37^. Disorder of phenylalanine metabolism s delays the process of phenylalanine translating into tyrosine and contributes to overbalanced levels of phenylalanine and tyrosine, and the equal phenomenon was also observed in the plasma of subjects in the psychological suboptimal health group in this study. Furthermore, researchers have shown that a higher level of phenylalanine would induce damage in the nervous system, resulting in hypokinesia, depression and psychogeny^38^. Previous research also suggested that phenylalanine was a large neutral amino acid that could affect 5-HT synthesis^39^,^40^. So we could deduce that a higher level of phenylalanine accompanying physical symptoms would indicate a state of psychological suboptimal health and imply that damages to the nervous system were in progress, and if it was ignored, mental disorder would emerge soon. In generally, valine and isoleucine are called branched-chain amino acids (BCAAs) because of their aliphatic side-chains. The decreased concentration of BCAAs in plasma could be an indication of the abnormal release of brain 5-HT that is highly related to central fatigue^41^,^42^, which is in conformity with common symptoms of psychological suboptimal health in clinical that easy to get fatigued and memory deterioration.

Also some metabolites at abnormality levels may be the precursor of neurotoxicity in nervous system, in this research, the major endogenous molecule we found was glutamine. As reported previously, glutamate is the primary excitatory neurotransmitter in the mammalian brain^43^. Through glia cells, glutamate is converted to glutamine and released into the extracellular fluid from which it is reabsorbed into presynaptic terminals and converted back to glutamate via the action of neuronal glutaminase. Glutamine and glutamate are inter-converted between neurons and astrocytes, which is necessary for glutamine homeostasis^44^. It induces neurotoxicity and is related to the neurobiology of depression if excessively released^45^,^46^. Also the increased level of glutamine in plasma may be a compensatory adaptation to counteract glutamate-induced neurotoxicity. This is in agreement with previous hypotheses^47^,^48^.

Individuals in state of psychological suboptimal health are struggling with the condition of lack of vitality in clinical, in most instances and the appearance may indicate that metabolic disturbance of energy is highly related the pathomechanism. Citrate, as a potential biomarker which is related to energy metabolism, is a dominant intermediate of the tricarboxylic acid cycle (TCA). The higher level of citrate in the plasma samples of the subjects in the state of psychological suboptimal health is indicative of TCA cycle dysfunction. Also levels of the BCAAs containing valine and isoleucine declined sharply, suggesting their consumption in large quantities for energy supply^49^, meanwhile isoleucine deficiency is marked by muscle tremors. Moreover an organism would be forced to produce ATP by anoxic respiration to adapt to the supply/demand imbalance because of deficient energy and the decreased level of glucose can be considered an indicator of the severity of the supply/demand imbalance. All the analysis of abnormal metabolic pathways energy related showed close relationship with clinical symptoms.

Loss of appetite, a common symptom of psychological suboptimal health, has shown contact with abnormalities in gut microflora. Trimethylamine N-oxide (TMAO) is an oxidation product of trimethylamine (TMA) and a common metabolite in animals and human. In particular, TMAO is biosynthesized endogenously from TMA, which is derived from choline obtained from dietary lecithin or dietary carnitine. Several previous clinical studies have indicated that depressed patients display a disturbance of gut microflora, including concentration changes of metabolites such as TMAO, DMA and dimethylglycine^50^. Previous research also demonstrated that plasma choline is derived from phosphorylcholine by phosphotransferase. TMA could be converted by choline via gut microbiota and then detoxified through flavin monooxygenase in the liver, forming TMAO^51^. Therefore, it is plausible that the state of psychological suboptimal health caused a disturbance in gut microbiota colonies.

Furthermore, we observed a higher level of N-acetyl-glycoproteins in the group of patients with psychological suboptimal health although most of the broad protein was eliminated by the method presented above and the resonances were suppressed by the CPMG pulse sequence^52^. Acetyl-glycoproteins are acute-phase proteins that can act as inflammation mediators^53^ and the levels of these proteins increase immediately in response to external or internal challenges such as infection, inflammation, and stress^49^ that are believed to be the cause of the state. Alterations in the levels of N-acetyl-glycoproteins may indicate that people have been suffering in an extreme environment and are developing psychological suboptimal health. This analysis would be the proof of close connection between N-acetyl-glycoproteins and extraneous factors leading to disease.

All of the analysis above would be the foundation and deep proof of the relationship among the metabolites and pathological mechanisms as well as incentives. These metabolic changes and the associated pathways provide insights into the mechanisms involved in the development and progression of psychological suboptimal health.

Furthermore, for the purpose of screening more representative biomarkers, methods of correlation analysis for selecting biomarkers as a biomarker panel and drug intervention for validating the close internal relations between the biomarker panel and the state were united. Then a biomarker panel containing phenylalanine, glutamine, tyrosine, citrate, N-acetyl-glycoproteins and TMAO was identified and high correlation with the state of psychological suboptimal was also demonstrated. As following, the ROC curve analysis for evaluation of clinical diagnosis ability was carried out. Small AUC of single one metabolite showed low diagnostic capability for the reason of small sample size or one metabolite cannot reflect comprehensively. But biomarker panel displayed the highest AUC (0.971) that show perfect diagnostic and recognition capability of psychological suboptimal health and would be used as an innovative diagnosis method.

Finally, although a biomarker panel was sought out using ^1^H-NMR metabolomics, but a large number of clinical samples should be collected and technologies of GC-MS and LC-MS should be used to quantify these metabolites of the biomarker panel for the ultimate goal that the biomarkers can be applied in clinical diagnosis.

## Materials and Methods

### Ethical statement

All control and psychological suboptimal health subjects provided informed consent prior to the collection of any data. This research was approved by the Ethical Committee of the First Hospital of Shanxi Medical University in Taiyuan and was conducted according to the principles expressed in the Declaration of Helsinki. Written informed consents from all recruited participants were acquired.

### Subjects and assessment

In this study, patients being the state of psychological suboptimal health (31–60 years) were filtrated from the traditional Chinese Medical Department of the First Affiliated Hospital of Shanxi Medical University as Baihe Dihuang Tang treatment group. Then age-and sex-matched mental health subjects were recruited to be the healthy controls. Briefly, patients were screened by items as follows:(1) totally scored ≥9 and ≤250 diagnosed by the scale of SCL-90; (2) cardinal symptom on the diagnostic criteria for deficiency of yin referring to the diagnosis curative standard of TCM disease; (3) not on any narcoleptic drugs within one year; (4) no mental disease, pregnancy, organic disease and allergic to TCM. The healthy controls should meet the standards: (1) score of SCL-90 should be at the point of 90; (2) no any previous history of neurological; (3) no systemic medical illness.

### Sample size calculation

In the design of clinical trials, the number of participants was determined by the manipulators and the participators were made up of 30 patients and 30 healthy controls. Through screening outpatients in the hospital and recruiting healthy volunteers for 2 months, 30 patients and 30 healthy controls were included into the trial through the assessment standard mentioned above.

Unfortunately, 8 patients were lost during the 4-week intervention with BDT with the potential reasons of the following: (1) medication cycle of 4 weeks was a little bit longer; (2) unable to endure the slow onset of TCM drug action; (3) not follow the doctor’s advice and take other drugs not allowed in the trial. Moreover, 7 healthy controls fell off for the possible reasons followed: (1) suffering from a cold, inflammation or other diseases at the point of collecting samples; (2) not want to take part in this trial continuously; (3) not get to the hospital because of some unexpected situation. So at the end of the trial, samples of 22 patients and 23 healthy controls were used for analysis.

### BDT preparation process and dosage

The medicinal plants used to prepare BDT decoction were purchased from the Chinese herbal medicine market in the city of An-guo, Hebei Province and authenticated by Professor Xue-mei Qin from Modern Research Center for Traditional Chinese Medicine, Shanxi University. The preparation was done in traditional Chinese Medical Department of the First Affiliated Hospital of Shanxi Medical University, where the standard machine and manipulators were performed according to the guidelines. Each dosage of BDT containing Lily bulb (30 g) and Rehmannia root (20 g) were soaked in water (1:8, w/v) for 30 min at room temperature and boiled for 1 h. The aqueous extract were filtered and concentrated to the volume of 200 mL, and then divided in two parts with the package automatically. The BDT was administrated to the patients with one dosage every day for 4 weeks and drinking or seafood was strictly prohibited in the case of the interference with this protocol.

This clinical work was performed strictly and correctly in the First Affiliated Hospital of Shanxi Medical University, which is a first-class hospital with national clinical trials research center of new drugs (GCP center). Also the hospital has ethics committee and this work had been permitted. The manipulators of the research have been engaged in clinical work for many years, specializing in the treatment of mental disorders and participated clinical trials of new drugs on many occasions. Experimental program had been designed by the manipulators and they ensured the standardization of the experimental process according to the Good Clinical Practice.

### Plasma sample collection

After the patients had fasted, 5 mL of venous blood was collected from all subjects in the psychological suboptimal health group into 10 mL heparin sodium tubes before and after 4 weeks of treatment. Blood was also collected from healthy controls before 4 weeks in the morning. Samples were centrifuged at 1250 × g for 15 min at 4 °C, after which each plasma sample was divided into equal aliquots and stored at −80 °C for subsequent analysis.

### Sample preparation

Plasma Samples were thawed at 0 °C in an ice-water mixture. First, 450 μl of plasma was mixed with 900 μl of analytical pure methanol, vortexed for 2 min, and then centrifuged at 16172 × g for 20 min at 4 °C to pellet proteins. After that, 1000 μl of supernatant was transferred into an EP tube. Another 900 μl of analytical pure methanol was added again, and the mixture was centrifuged at 16172 × g for 20 min at 4 °C for outright protein removal. Finally, a total of 1800 μl of supernatant was dried under nitrogen gas, and the dried samples were completely dissolved in 600 μl phosphate buffer solution in 100% D2O (0.2 M Na2HPO4/NaH2PO4, pD = 7.4) containing TSP (0.025%) to minimize chemical shift variations. The samples were then centrifuged (16172 × g, 10 min, at 4 °C) to eliminate any precipitates, and 550 μl of supernatant was transferred into 5 mm NMR tubes for NMR analysis^47^.

### Metabolic profiling data acquisition

A Bruker 600 MHz AVANCE III NMR spectrometer (Bruker Biospin, Rheinstetten, Germany) was used to receive the ^1^H-NMR spectra of plasma samples, operating at a ^1^H frequency of 600.13 MHz and a temperature of 298 K. A one-dimensional (1D) Carr-Purcell-Merboom-Gill (CPMG, RD–90− (τcp−180−τcp) -acquisition) with water suppression and a total spin-spin relaxation delay of 320 ms was set for the plasma analysis. The ^1^H NMR spectrum for each sample consisted of 64 scans requiring 2.7 min of acquisition time with the following parameters: spectral width = 12019.2 Hz, spectral size = 65536 points, pulse width(90) = 14.0 μs, and relaxation delay (RD) = 1.0 s. FIDs were Fourier transformed with LB = 0.3 Hz.

For a good signal dispersion and visualization, two-dimensional (2D) NMR spectra for the selected samples were also recorded using a 298 k on Bruker 600 MHz AVANCE III NMR spectrometer, including 1H–1H correlation spectroscopy (COSY) and 1H–13C heteronuclear multiple quantum coherence (HMQC). The 2D 1H-1H COSY experiments were acquired in magnitude mode (Bruker pulse sequence cosygpqf) at 600 MHz with 2k data points in F2 and 256 increments in F1, using spectral widths of 6602.1 and 6601.5 Hz in both dimensions. A total of 25 transients were collected with an acquisition time of 0.155 s. The relaxation delay was 1.5 s, the 90 pulse width was 14.0 μs, and the receiver gain 203. And also the 2D 1H-13C HMQC experiments were acquired in magnitude mode (Bruker pulse sequence hmqcgpqf) at 600 MHz with 1 k data points in F2 and 256 increments in F1, using a spectral width of 6602.1 Hz in ^1^H dimension and 36219.4 Hz in the ^13^C dimension. A total of 110 transients were collected with an acquisition time of 0.078 s. The relaxation delay was 1.5 s, the 90 pulse width was 14.0 μs, and the receiver gain 203.

### NMR data preprocessing

The baseline and phase pretreatment of the acquired 1H NMR files were set manually with MestReNova software (Mestrelab Research, Santiago de Compostella, Spain). All of the spectra were referenced to the chemical shift of TSP located at δ 0.00 ppm. After the regions of δ 4.70–5.20 and δ 3.34–3.37 ppm were removed to eliminate the influence of water and methanol, the spectra were segmented at δ 0.01 intervals across the chemical shift range of 0.5 to 9.00 ppm. To reduce significant concentration differences between the samples, the integral values from each spectrum were normalized to a sum of all of the integrals in a spectrum, and date matrices were constructed for further multivariate analysis^54^,^55^.

### Data analysis

Prior to statistical analysis, all resulting integral data from ^1^H-NMR metabolomics analysis were imported into SIMCA-P13.0 (Umetrics, Sweden) for multivariate analysis. Partial least squares discrimination analysis (PLS-DA) was conducted to distinguish different groups in a supervised manner. Parameters for model fitness (R^2^) and predictive ability (Q^2^) with leave-one-out cross validation and the response of the permutation test (200 cycles) should be used to evaluate whether the model is established or not because of the small number of samples^56^. Furthermore, a supervised pattern recognition approach known as an orthogonal projection to latent structures discriminant analysis (OPLS-DA) was used to improve the classification of the different groups while screening biomarkers. With an aim to discover the potential variables contributing to the differentiation, we generated an S-plot for the OPLS-DA model used to define metabolites significantly contributing to the separation of groups. On the basis of the variable importance in the project (VIP) threshold of 1 (VIP ≥ 1.00), a number of metabolites responsible for the difference in metabolic profiles of different groups could be obtained. In parallel, the metabolites identified by the OPLS-DA were validated at a univariate level using *t*-test (SPSS 17.0) with the critical *p* value set to 0.05 in order to detect the main metabolites that were significantly different leading to the class discrimination.

A system statistical metabolic correlation analysis was further applied to display the relationships between these certain metabolite integrals^57^. Metabolite intensities relative to the sum of the total spectral integral were used as variables, and Spearman’s correlation coefficient was calculated among those variables using Java. An absolute value of the correlation coefficient indicates a statistically significant relationship among these potential biomarkers. Positive values masked in the pixel map are shown with red colors, and negative values are indicated with blue colors. A receiver operating characteristic (ROC) curves was carried out to further evaluate the performance of the metabolites selected by *t*-test in clinical diagnosis. The area under the curve (AUC) was used to evaluate diagnostic psychological suboptimal health state values in the clinic.

## Additional Information

**How to cite this article**: Tian, J.-s. *et al.* Discovery, screening and evaluation of a plasma biomarker panel for subjects with psychological suboptimal health state using ^1^H-NMR-based metabolomics profiles. *Sci. Rep.* **6**, 33820; doi: 10.1038/srep33820 (2016).

## Supplementary Material

Supplementary Information

## Figures and Tables

**Table 1:** General characteristic of the participants at baseline and at the end of the Baihe Dihuang Tang intervention (4 weeks) and the healthy controls.

	Psychological suboptimal health		Healthy controls
	Before 4 weeks	After 4 weeks	
Sample size	22	22	23
Sex (M/F)	4/18	4/18	5/18
Age (year)	48.7 ± 5.3	48.7 ± 5.3	49.3 ± 4.6
SCL-90	143.9 ± 22.6	112.4 ± 11.5**	90

M: male; F: Female; SCL-90: The Symptom Checklist 90.

**P < 0.01 before and after 4 weeks.

**Table 2:** Peak attribution of the main marked metabolites in ^1^H-NMR spectra of plasma samples.

Key	Metabolites	Moieties	δ^1^H/ppm and multiplicity/Hz	Key	Metabolites	Moieties	δ^1^H/ppm and multiplicity/Hz
1	2-OH-butyrate	γCH_3_	0.90 (t, 7.47)	17	Isoleucine	δCH_3_ γ’CH_3_	0.94 (t, 7.4) 1.02 (d, 7.0)
2	3-OH-butyrate	γCH_3_ αCH	1.20 (d, 6.60) 4.15 (m)	18	Lactate	βCH_3_ αCH	1.33 (d, 6.9) 4.12 (q, 6.9)
3	Acetate	βCH_3_	1.93 (s)	19	Leucine	δCH_3_ αCH_2_	0.96 (d) 3.73 (m)
4	Acetoacetate	CH_3_ CH	2.28 (s) 3.48 (s)	20	Lipids	CH_3_ (CH_2_)_n_	0.84 (t) 1.28 (m)
5	Alanine	βCH_3_ CH	1.48 (d, 7.3) 3.77 (m)	21	Methionine	γCH_2_ S-CH_3_	2.62 (t, 7.58) 2.14 (s)
6	Betaine	N(CH_3_)_3_ CH_2_	3.27 (m) 3.89 (s)	22	Methylamine	CH_3_	2.61 (s)
7	Carnitine	N(CH_3_)_3_	3.21 (s)	23	Methanol	CH_3_	3.36 (s)
8	Choline	N(CH_3_)_3_	3.20 (s)	24	N-acetyl-glycoproteins	CH_3_	2.04 (s)
9	Citrate	Half CH_2_ Half CH_2_	2.54 (d, 16.1) 2.65 (d, 16.2)	25	Phosphatidylcholine	N(CH_3_)_3_	3.22 (s)
10	Creatine	N-CH_3_ CH_2_	3.93 (s) 3.04 (s)	26	Phenylalanine	2 and 6-CH 3 and 5-CH	7.33 (m) 7.42 (m)
11	Cysteine	CH CH_2_	3.97 (dd) 3.06 (m)	27	Proline	αCH_2_ βCH_2_	1.99 (m) 2.34 (m)
12	Glutamine	αCH βCH_2_	2.16 (m) 2.45 (m)	28	Pyruvate	CH_3_	2.38 (m)
13	Glutamate	βCH_2_ γCH_2_	2.07 (m) 2.35 (m)	29	Trimethylamine oxide	CH_3_	3.26 (m)
14	Glycine	αCH_2_	3.57 (s)	30	Tyrosine	3 and 5-CH 2 and 6-CH	6.90 (m) 7.19 (m)
15	glycerol	CH_2_ CH	3.67 (m) 3.78 (m)	31	Valine	γCH_3_ γ’CH_3_	0.99 (d, 7.1) 1.05 (d, 7.0)
16	Histidine	2-CH 4-CH	7.68 (s) 7.10 (s)	32	Glucose	C_1_H	5.22 (d, 3.7) 4.64 (d, 8.0)

^a^s: singlet, d: doublet, t: triplet, q: quartet, m: multiplet, dd: doublet of doublet.

**Table 3:** Metabolites selected as biomarkers characterized in plasma profile and their change trend after Baihe Dihuang Tang treatment.

No.	Metabolites	Shift chemical^a^	Patients *vs.* Control^b^	*P* value	Treated *vs.* before Treatment^b^	*P* value	Metabolism Pathway
1	valine	1.00 (d) 1.05 (d)	↓	2.26E-03*	↑	3.02E-05*	Amino acid metabolism
2	Isoleucine	0.94 (t) 1.02 (d)	↓	3.04E-02*	↑	2.09E-01	Amino acid metabolism
3	Glutamine	2.45 (m) 2.16 (m)	↑	2.64E-04*	↓	4.56E-03*	Amino acid metabolism
4	Citrate	2.54 (d)	↑	1.13E-02*	↓	7.59E-02	TCA cycle
5	Glucose	4.66 (d)	↓	1.99E-03*	↑	2.06E-01	Glucose metabolism
6	Trimethylamine oxide	3.26 (s)	↑	8.76E-03*	↓	4.99E-10*	Methylamine metabolism
7	N-acetyl-glycoproteins	2.04 (s)	↑	1.13E-02*	↓	6.88E-01	inflammatory responses
8	Tyrosine	6.90 (m) 7.19 (m)	↑	9.15E-04*	↓	1.63E-01	Amino acid metabolism
9	Phenylalanine	7.36 (m) 7.42 (m)	↑	1.22E-03*	↓	8.60E-03*	Amino acid metabolism

^a^Multiplicity definitions: s, singlet; d, doublet; t, triplet; m, multiplet.

^b^Metabolites with“↑/↓” means increased/decreased, “*” means dates significant differences.

**Table 4:** Area under the curves of the biomarker combinations.

Biomarkers	Area	Std. Error	Asymptotic Sig.	Asymptotic 95% Confidence Interval	
				Lower Bound	Upper Bound
A	0.814	0.068	0.000	0.680	0.948
A + B	0.882	0.053	0.000	0.778	0.987
A + B + C	0.880	0.051	0.000	0.781	0.979
A + B + C + D	0.890	0.048	0.000	0.797	0.984
A + B + C + D + E	0.924	0.040	0.000	0.845	1.002
A + B + C + D + E + F	0.971	0.020	0.000	0.931	1.011

A, Citrate; B, Glutamine; C, Tyrosine; D, Phenylalanine; E, TMAO; F, N-acetyl-glycoproteins.
