# Supplementary material for: Discovery, screening and evaluation of a plasma biomarker panel for subjects with psychological suboptimal health state using 1H-NMR-based metabolomics profiles
Source: Sci Rep. 2016 Sep 21;6:33820. doi: 10.1038/srep33820 (PMC5030673; doi:10.1038/srep33820)
Supplement: Supplementary Information [file srep33820-s1.doc]

**Discovery, screening and evaluation of a plasma biomarker panel for subjects with psychological suboptimal health state using ^1^H-NMR-based metabolomics profiles**

Jun-sheng Tian^1^, Xiao-tao Xia^1,2^, Yan-fei Wu^3^, Lei Zhao^1^, Huan Xiang^4^, Guan-hua Du^5^, Xiang Zhang^6^, Xue-mei Qin^1,*^

^1^Modern Research Center for Traditional Chinese Medicine of Shanxi University, Taiyuan 030006, P. R. China;

^2^ College of Chemistry and Chemical Engineering of Shanxi University, Taiyuan 030006，P. R. China

^3^ Department of traditional Chinese medicine, First Hospital of Shanxi Medical University, Taiyuan 030001, P. R. China

^4^ Physical Education Departments of Shanxi University, Taiyuan 030006, P. R. China;

^5^ Institute of Materia Medica, Chinese Academy of Medical Sciences & Peking Union Medical College, Beijing 100050, P. R. China

^6^ Department of Chemistry of University of Louisville, Louisville, KY40292 United States

^*^Correspondence and requests for materials should be addressed to Xue-mei Qin ([qinxm@sxu.edu.cn](mailto:qinxm@sxu.edu.cn)).


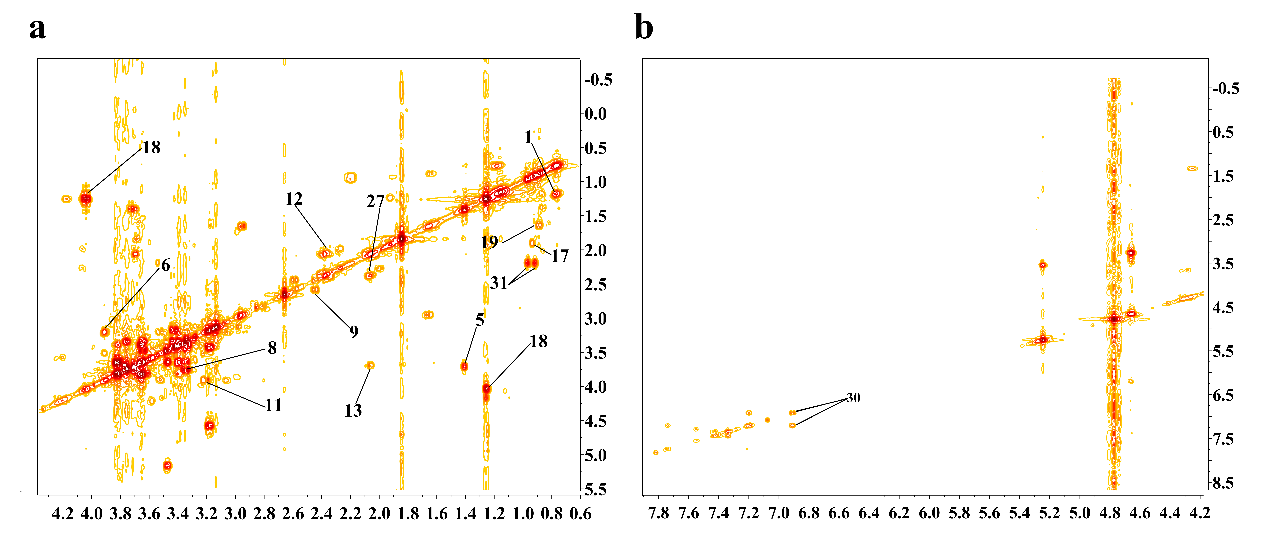
For a good signal dispersion and visualization, two-dimensional (2D) NMR spectra for the selected samples were also recorded using a 298k on Bruker 600 MHz AVANCE III NMR spectrometer, including 1H–1H correlation spectroscopy (COSY) and 1H–13C heteronuclear multiple quantum coherence (HMQC). The 2D 1H-1H COSY experiments were acquired in magnitude mode (Bruker pulse sequence cosygpqf) at 600 MHz with 2k data points in F2 and 256 increments in F1, using spectral widths of 6602.1 and 6601.5 Hz in both dimensions. A total of 25 transients were collected with an acquisition time of 0.155 s. The relaxation delay was 1.5 s, the 90 pulse width was 14.0 µs, and the receiver gain 203. And also the 2D 1H-13C HMQC experiments were acquired in magnitude mode (Bruker pulse sequence hmqcgpqf) at 600 MHz with 1k data points in F2 and 256 increments in F1, using a spectral width of 6602.1 Hz in 1H dimension and 36219.4 Hz in the 13C dimension. A total of 110 transients were collected with an acquisition time of 0.078 s. The relaxation delay was 1.5 s, the 90 pulse width was 14.0 µs, and the receiver gain 203.

**Figure S1** ^1^H−^1^H COSY 600 MHz spectra of human plasma metabolites with protein removal using methanol. Identified metabolites:1, 2-OH-butyrate; 5, Alanine; 6, Betaine; 7, Carnitine; 8, Choline; 9, Citrate; 11, Cysteine; 12, Glutamine; 13, Glutamate; 17, Isoleucine; 18, Lactate; 19,Leucine; 27, Proline; 30,Tyrosine; 31,Valine.


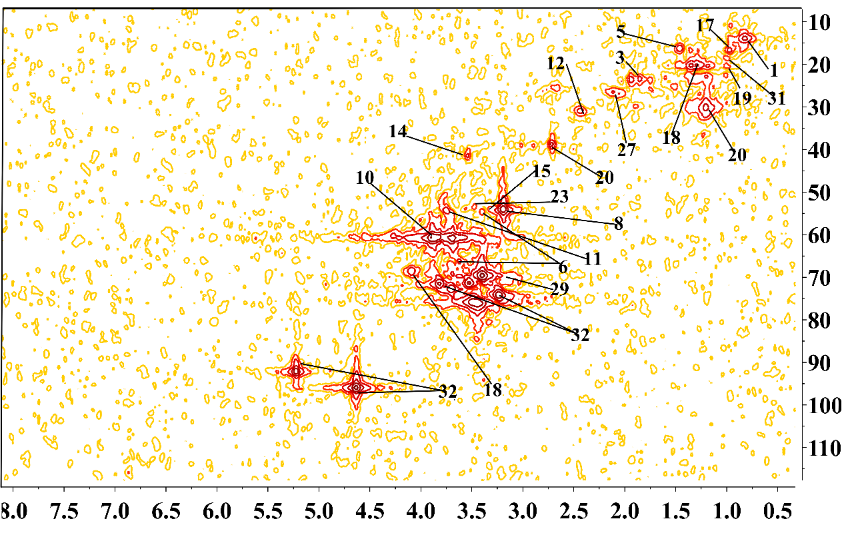


**Figure S2** ^1^H−^13^C HSQC 600 MHz spectrum of human plasma metabolites with protein removal using methanol. Identified metabolites:1, 2-OH-butyrate; 3, Acetate; 5, Alanine; 6, Betaine; 8, Choline; 10, Creatine; 11, Cysteine; 12, Glutamine; 14, Glycine; 15, Glycerol; 17, Isoleucine; 18, Lactate;19, Leucine; 20, Lipids; 23, Methanol; 27, Proline; 29, Trimethylamine oxide;31, Valine; 32, Glucose.

**
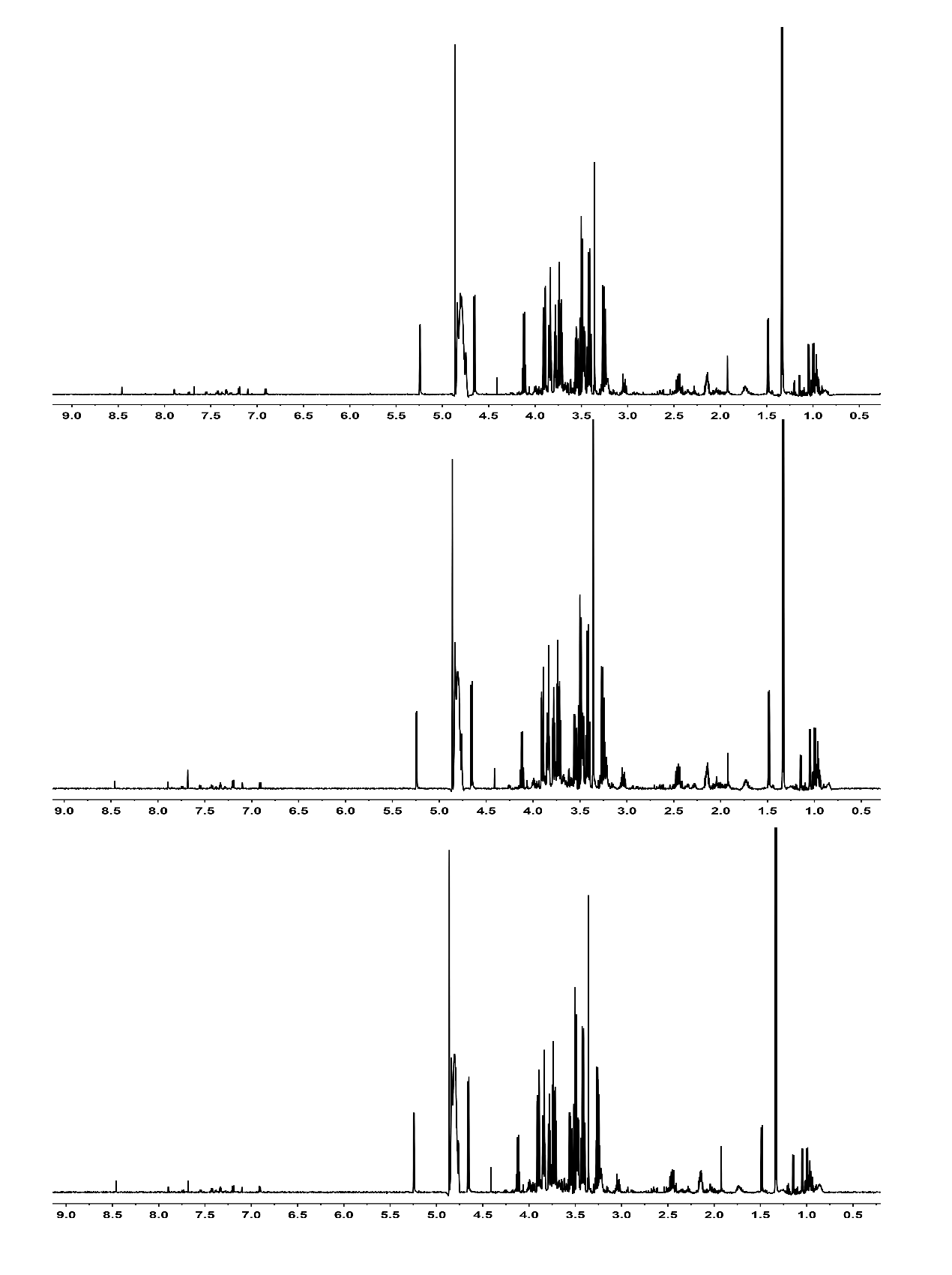
**

**Figure S3** Typical ^1^H NMR plasma spectra of healthy controls

**
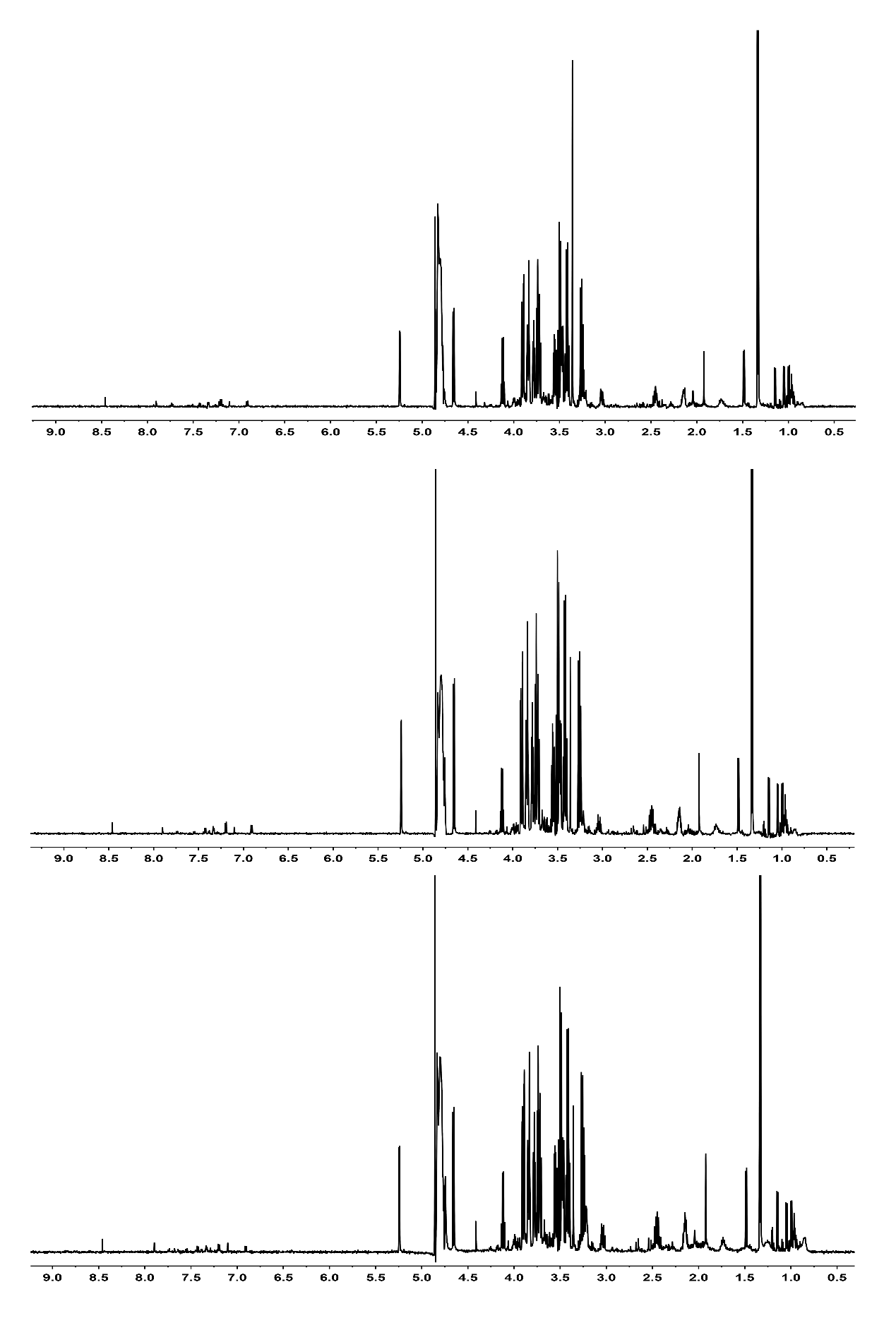
**

**Figure S4** Typical ^1^H NMR plasma spectra of Baihe Dihuang Tang group

**
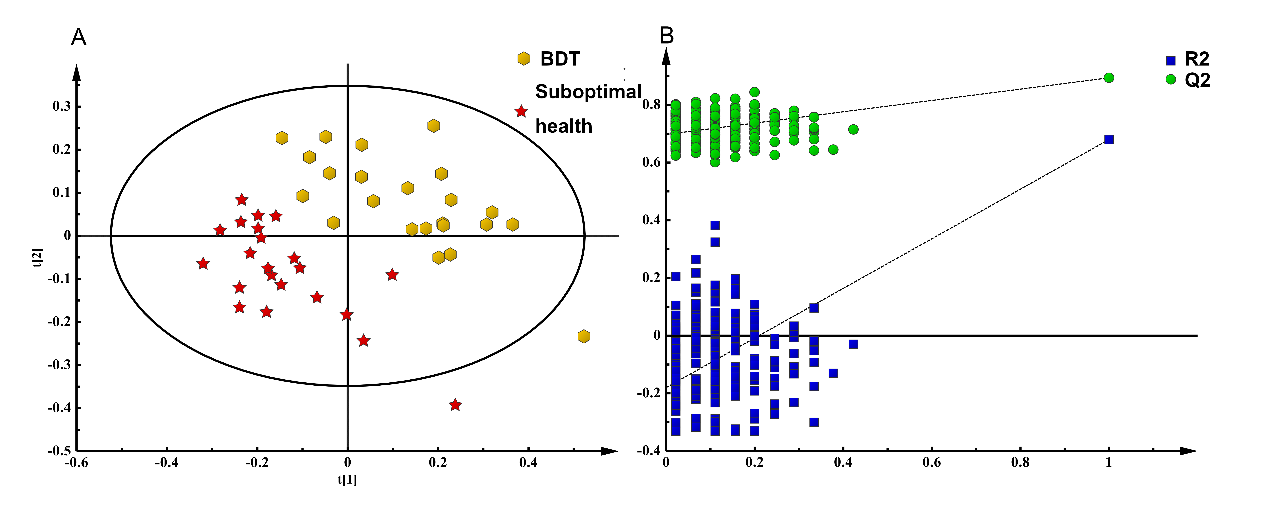
**

**Figure S5** Pattern recognition with Simca-p13.0. The PLS-DA score plot derived from ^1^H NMR plasma spectra of psychological suboptimal health group compared with BDT group (A). The PLS-DA validation plot (permutation number: 200) pair-wise comparison of plasma from psychological suboptimal health group and BDT group (B).

**
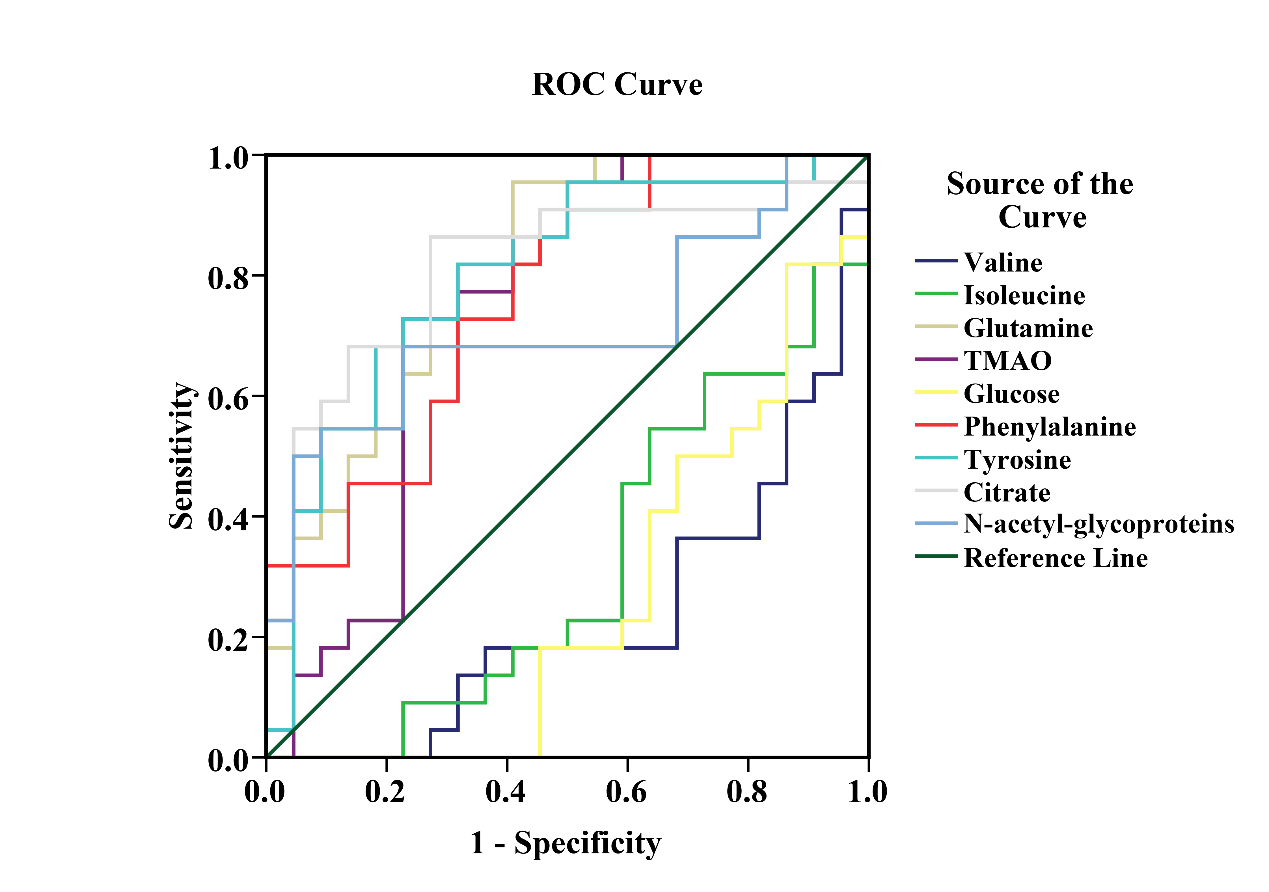
**

**Figure S6** ROC curves of different potential biomarkers for diagnosis between groups of psychological suboptimal health and healthy controls.

**Table S1** The parameters indicating the model quality of OPLS-DA

|  | OPLS-DA model | | |  |
| --- | --- | --- | --- | --- |
| Patients  *vs.* Healthy controls  Patients *vs.* Treatment | R^2^X | R^2^Y | Q^2^ | CV-ANOVA(*p*<0.05) |
|  | 0.541 | 0.949 | 0.778 | 2.19E-009 |
|  | 0.403 | 0.894 | 0.741 | 1.68E-009 |

**Table S2 Area under the curves of the different potential biomarkers**

| Biomarkers | Area | Std. Error | Asymptotic Sig. | Asymptotic 95% Confidence Interval | |
| --- | --- | --- | --- | --- | --- |
|  |  |  |  | Lower Bound | Upper Bound |
| Valine | 0.233 | 0.072 | 0.002 | 0.092 | 0.375 |
| Isoleucine | 0.318 | 0.081 | 0.039 | 0.160 | 0.476 |
| Glutamine | 0.808 | 0.065 | 0.000 | 0.680 | 0.935 |
| TMAO | 0.746 | 0.079 | 0.005 | 0.591 | 0.900 |
| Glucose | 0.264 | 0.076 | 0.007 | 0.115 | 0.414 |
| Phenylalanine | 0.762 | 0.071 | 0.003 | 0.623 | 0.902 |
| Tyrosine | 0.802 | 0.068 | 0.001 | 0.668 | 0.936 |
| N-acetyl-glycoproteins | 0.713 | 0.081 | 0.016 | 0.554 | 0.872 |
| Citrate | 0.814 | 0.068 | 0.000 | 0.680 | 0.948 |
